# Supplementary material for: Lifestyle and Health-Related Quality of Life Relationships Concerning Metabolic Disease Phenotypes on the Nutrimdea Online Cohort
Source: Int J Environ Res Public Health. 2022 Dec 31;20(1):767. doi: 10.3390/ijerph20010767 (PMC9819172; doi:10.3390/ijerph20010767)
Supplement: Supplementary file 1 [file ijerph-20-00767-s001.zip › ijerph-2052375-supplementary.pdf]

TABLE S1. Phenotypic characteristics, lifestyle, HRQoL and Obesogenic Score of the participants in the NUTRiMDEA study stratified by physical activity, diet and type of survey

	Open Survey		Rewarded Survey				
	**Low PA**	**High PA**	**Low PA**	**High PA**	**P for PA**	**P for Survey**	**P of interaction**
**n**	4673	4960	1903	1887			
**Weight, mean (SE)**	70.17 (0.16)^{b}	68.76 (0.15)^{a}	72.24 (0.32)^{c}	70.53 (0.32)^{b}	0.063	<0.001	0.528
**Height, mean (SE)**	170. 2 (0.1)^{ab}	170.3 (0.09)^{b}	169.7 (0.19)^{a}	169.9 (0.19)^{ab}	<0.001	<0.001	0.809
**BMI, mean (SE)**	24.11 (0.05)^{b}	23.58 (0.05)^{a}	24.99 (0.1)^{c}	24.34 (0.1)^{b}	<0.001	<0.001	0.408
**Trousers size, mean (SE)**	40.53 (0.06)^{b}	39.91 (0.05)^{a}	41.54 (0.11)^{c}	41.21 (0.11)^{c}	<0.001	<0.001	0.090
**MDS14, mean (SE)**	7.45 (0.03)^{c}	8.14 (0.03)^{d}	6.26 (0.06)^{a}	7.06 (0.06)^{b}	<0.001	<0.001	0.221
**PA light (min/week), mean (SE)**	131.0 (2.30)^{a}	286.0 (2.19)^{b}	131.0 (4.49)^{a}	302.0 (4.52)^{c}	<0.001	0.191	<0.05
**PA moderate (min/week), mean (SE)**	33.2 (1.26)^{a}	110.4 (1.20)^{b}	34.6 (2.46)^{a}	133.9 (2.47)^{c}	<0.001	<0.001	<0.001
**PA intense (min/week), mean (SE)**	41.8 (1.48)^{a}	185.7 (1.41)^{b}	33.7 (2.89)^{a}	185.5 (2.91)^{b}	<0.001	<0.01	0.077
**Total PA (METs-min/week), mean (SE)**	900.0 (12.5)^{a}	2872.0 (11.9)^{b}	839.0 (24.4)^{a}	3016.0 (24.5)^{c}	<0.001	0.633	<0.001
**PCS12, mean (SE)**	53.59 (0.1)^{c}	55.73 (0.09)^{d}	50.02 (0.19)^{a}	52.03 (0.20)^{b}	<0.001	<0.001	0.663
**MCS12, mean (SE)**	42.5 (0.16)^{b}	45.2 (0.15)^{c}	41.3 (0.31)^{a}	42.9 (0.32)^{b}	<0.001	0.371	<0.05
**Obesogenic Score, mean (SE)**	1.86 (0.01)^{c}	1.41 (0.01)^{b}	1.83 (0.03)^{c}	1.29 (0.03)^{a}	<0.001	0.130	<0.05
	**Low MDS14**	**High MDS14**	**Low MDS14**	**High MDS14**	**P for MDS14**	**P for Survey**	**P of interaction**
**n**	5829	3949	3332	590			
**Weight, mean (SE)**	70.05 (0.14)^{b}	68.55 (0.18)^{a}	71.61 (0.25)^{c}	70.60 (0.52)^{bc}	<0.001	<0.001	0.422
**Height, mean (SE)**	170.2 (0.09)^{b}	170.5 (0.11)^{b}	169.6 (0.15)^{a}	170.2 (0.31)^{ab}	0.090	<0.001	0.472
**BMI, mean (SE)**	24.08 (0.05)^{b}	23.47 (0.06)^{a}	24.78 (0.08)^{c}	24.27 (0.16)^{b}	<0.001	<0.001	0.610
**Trousers size, mean (SE)**	40.31 (0.05)^{b}	40.08 (0.06)^{a}	41.43 (0.09)^{c}	41.13 (0.18)^{c}	<0.001	<0.001	0.736
**MDS14, mean (SE)**	6.60 (0.02)^{b}	9.79 (0.02)^{d}	6.00 (0.03)^{a}	9.61 (0.06)^{c}	<0.001	<0.001	<0.001
**PA light (min/week), mean (SE)**	194.0 (2.28)^{a}	237.0 (2.83)^{b}	206.0 (3.95)^{a}	255.0 (8.32)^{b}	<0.001	0.073	0.474
**PA moderate (min/week), mean (SE)**	65.8 (1.22)^{a}	85. 6 (1.52)^{b}	80.7 (2.12)^{b}	99.8 (4.46)^{c}	<0.001	<0.001	0.891
**PA intense (min/week), mean (SE)**	104.0 (1.62)^{a}	137.0 (2.0)^{b}	104.0 (2.80)^{a}	142.0 (5.89)^{b}	<0.001	0.101	0.411
**Total PA (METs-min/week), mean (SE)**	1740 (17.4)^{a}	2220 (21.6)^{c}	1836 (30.2)^{b}	2381 (63.6)^{c}	<0.001	0.228	0.381
**PCS12, mean (SE)**	54.31 (0.09)^{c}	55.37 (0.11)^{d}	50.52 (0.15)^{a}	52.93 (0.33)^{b}	<0.001	<0.001	<0.001
**MCS12, mean (SE)**	43.0 (0.14)^{b}	45.3 (0.18)^{c}	41.7 (0.25)^{a}	44.4 (0.52)^{c}	<0.001	<0.01	0.522
**Obesogenic Score, mean (SE)**	1.72 (0.01)^{d}	1.47 (0.02)^{b}	1.61 (0.02)^{c}	1.27 (0.05)^{a}	<0.001	<0.001	0.110

High or low MDS14 was determined with the median. Open Survey refers to a sample of users who completed the survey online for open. Rewarded Survey refers to a sample of users of different payment platforms that completed the survey. P-values for two way analysis of variance (ANOVA) with sidak post-hoc test between groups. Adjusted by type of survey, HRQoL (PCS12 and MCS12) age and sex. ^{abcd} means with different superscript are statistically different (threshold significance was set at $p<0.05$).

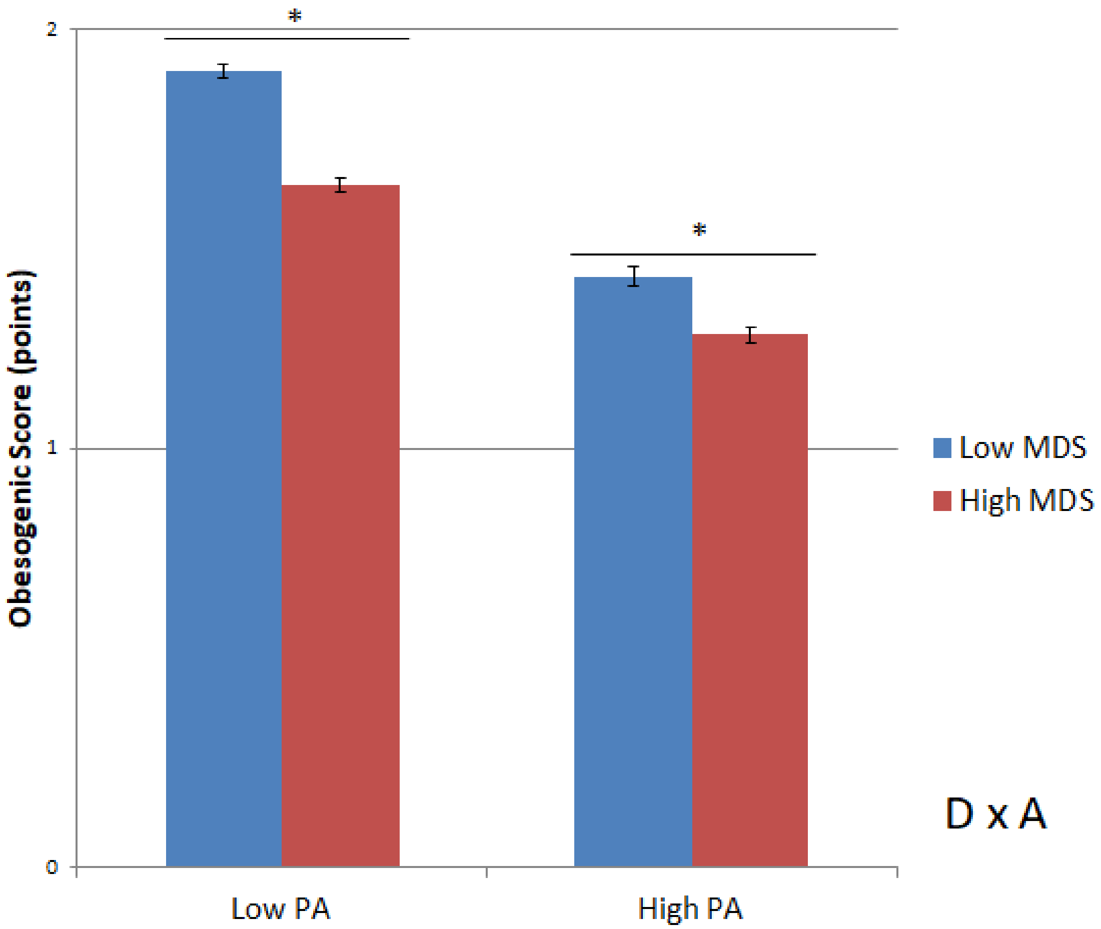

Figure S1. Obesogenic Score (mean±SE) depending on dichotomous stratification into low and high physical activity (Low and High PA) (METs-min/week) and low and high Mediterranean diet Score (Low and High MDS). D x A means interaction between MDS14 and physical activity ($p<0.001$). Threshold significance was set at $p<0.05$. *$p<0.05$. P-values for two way analysis of variance (ANOVA) with sidak post-hoc test between groups. Adjusted by type of survey, HRQoL (PCS12 and MCS12), age, sex and education.

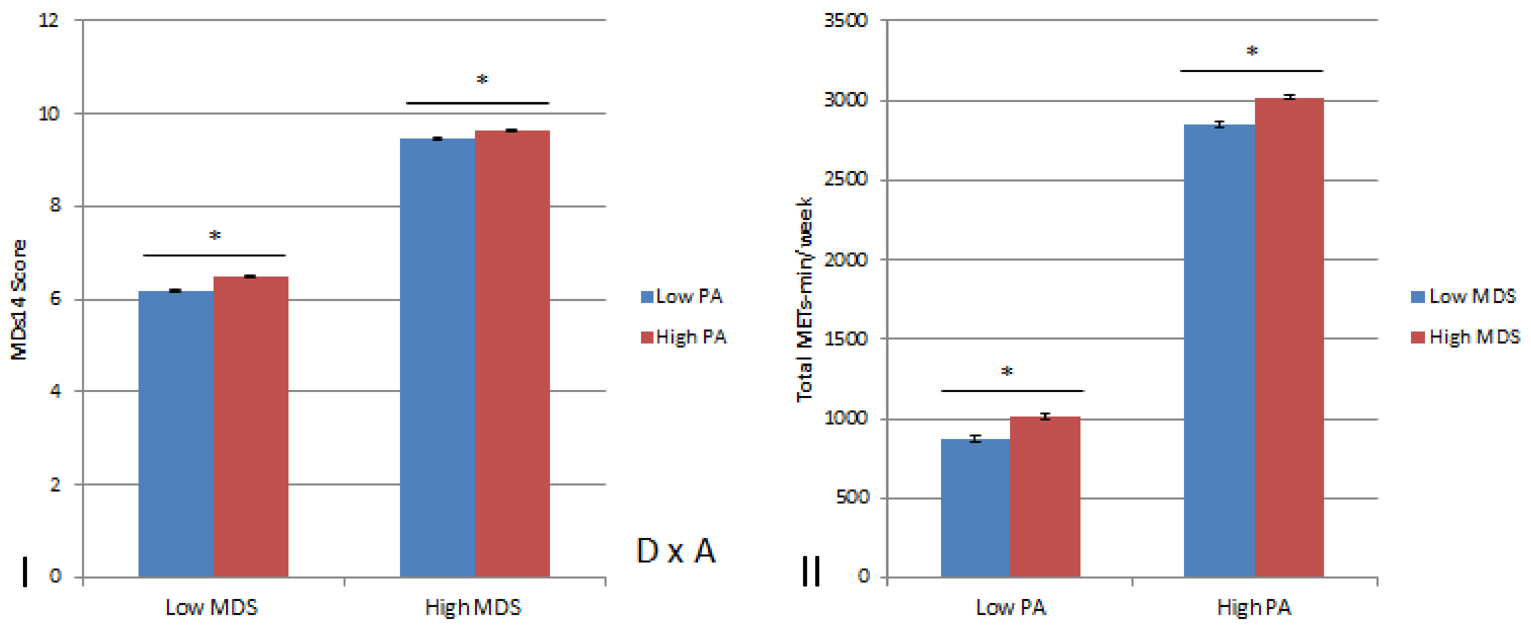

Figure S2. Threshold significance was set at p<0.05. *p<0.05. **I.** Mediterranean diet score (MDS14 score) (mean±SE) depending on dichotomous stratification into low and high physical activity (Low and High PA) and low and high Mediterranean diet Score (Low and High MDS). D x A means interaction between MDS14 and physical activity (p<0.01). P-values for two way analysis of variance (ANOVA) with sidak post-hoc test between groups. Adjusted by type of survey, HRQoL, age and sex. **II**. Level of total physical activity (Total METs-min/week) (mean±SE) depending on dichotomous stratification into low and high Mediterranean diet Score (Low and High MDS) and low and high physical activity (Low and High PA). P-values for two way analysis of variance (ANOVA) with sidak post-hoc test between groups. Adjusted by type of survey, HRQoL, age and sex.
